# Factors associated with disease progression in patients with atrial fibrillation and heart failure anticoagulated with rivaroxaban

**DOI:** 10.1002/clc.24189

**Published:** 2023-11-29

**Authors:** Nicolás Manito, José María Cepeda‐Rodrigo, Nuria Farré, Miguel Castillo Orive, Enrique Galve, Javier Jiménez‐Candil, José M. García‐Pinilla, Eduardo Sebastián López Sánchez, Carles Rafols, Juan José Gómez Doblas

**Affiliations:** ^1^ Cardiology Department Hospital Universitario de Bellvitge Barcelona Spain; ^2^ Department of Internal Medicine Hospital Vega Baja Orihuela Spain; ^3^ Cardiology Department Hospital del Mar Barcelona Spain; ^4^ Cardiology Department Hospital Universitario Ramón y Cajal Madrid Spain; ^5^ Consulta cardiología Galve Basilio Barcelona Spain; ^6^ Cardiology Department IBSAL‐Hospital Universitario de Salamanca, Universidad de Salamanca, CIBER‐CV Salamanca Spain; ^7^ Cardiology Department Hospital Universitario Virgen de la Victoria Málaga Spain; ^8^ Instituto de Investigación Biomédica de Málaga‐Plataforma BIONAND Málaga Spain; ^9^ Ciber‐Cardiovascular Instituto de Salud Carlos III Madrid Spain; ^10^ Department of Medicine and Dermatology Universidad de Málaga Málaga Spain; ^11^ Clínica Cardiología Vera Almería Spain; ^12^ Medical Department Bayer Hispania Barcelona Spain

**Keywords:** anticoagulation, atrial fibrillation, direct oral anticoagulant, heart failure, rivaroxaban, worsening heart failure

## Abstract

**Background:**

Patients with atrial fibrillation (AF) and heart failure (HF) have a high risk of thromboembolism and other outcomes and anticoagulation is recommended.

**Hypothesis:**

This study was aimed to explore the risk factors associated with HF worsening in patients with AF and HF taking rivaroxaban in Spain.

**Methods:**

Multicenter, prospective, observational study that included adults with AF and chronic HF, receiving rivaroxaban ≥4 months before entering. HF worsening was defined as first hospitalization or emergency visit because of HF exacerbation.

**Results:**

A total of 672 patients from 71 Spanish centers were recruited, of whom 658 (97.9%) were included in the safety analysis and 552 (82.1%) in the per protocol analysis. At baseline, mean age was 73.7 ± 10.9 years, 64.9% were male, CHA_2_DS_2_‐VASc was 4.1 ± 1.5, HAS‐BLED was 1.6 ± 0.9% and 51.3% had HF with preserved ejection fraction. After 24 months of follow‐up, 24.9% of patients developed HF worsening, 11.6% died, 2.9% had a thromboembolic event, 3.1% a major bleeding, 0.5% an intracranial bleeding and no patient had a fatal hemorrhage. Older age, the history of chronic obstructive pulmonary disease, the previous use of vitamin K antagonists, and restrictive or infiltrative cardiomyopathies, were independently associated with HF worsening. Only 6.9% of patients permanently discontinued rivaroxaban treatment.

**Conclusions:**

Approximately one out of four patients with HF and AF treated with rivaroxaban developed a HF worsening episode after 2 years of follow‐up. The identification of those factors that increase the risk of HF worsening could be helpful in the comprehensive management of this population.

## Graphical abstract

Multicenter, prospective, observational study that included 672 adults from 71 Spanish centers with AF and chronic HF, receiving rivaroxaban. At baseline, mean age was 73.7 ± 10.9 years, 64.9% were male, CHA_2_DS_2_‐VASc was 4.1 ± 1.5, HAS‐BLED was 1.6 ± 0.9% and 51.3% had HF with preserved ejection fraction. After 24 months of follow‐up, 24.9% of patients developed HF worsening, 11.6% died, 2.9% had a thromboembolic event, 3.1% a major bleeding, 0.5% an intracranial bleeding and no patient had a fatal hemorrhage. Older age, the history of chronic obstructive pulmonary disease, the previous use of vitamin K antagonists, and restrictive or infiltrative cardiomyopathies, were independently associated with HF worsening. Only 6.9% of patients permanently discontinued rivaroxaban treatment.

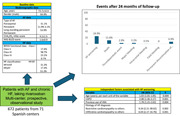

## 1 INTRODUCTION

Heart failure (HF) is a major healthcare problem.^1^ HF is associated with great morbidity and mortality.^2^ Importantly, HF hospitalization rates are increasing over time.^3^ In addition, it has been reported that one in six patients with HF with reduced ejection fraction may develop worsening HF within 18 months after HF diagnosis.^4^ A recent Spanish study has shown that nearly 30% of patients are hospitalized for HF within 1 year after the diagnosis, with a mortality rate of 8% during hospitalization.^5^ Therefore, worsening HF is a common condition, that can occur in different clinical settings (i.e., hospitalization, emergency department, outpatient). Identifying factors associated with worsening HF is warranted to reduce HF burden.^6^

It has been estimated that over 35% of patients with atrial fibrillation (AF) are also diagnosed with HF. This is not surprising, since HF and AF share common risk factors and mechanisms.^7^, ^8^ The concomitance of both conditions markedly worsens the prognosis.^9^ Patients with AF and HF have a high risk of thromboembolism, and anticoagulation is recommended.^10^

Although in the last years some studies have analyzed the risk factors for HF worsening, most of them have investigated all‐cause mortality associated with clinical characteristics of HF patients,^11^, ^12^, ^13^ only few studies have focused on variables associated with HF hospital admissions,^14^, ^15^ and in patients mainly taking vitamin K antagonists.^16^ Therefore, it seems necessary to determine those factors predicting worsening HF in AF patients treated with anticoagulant agents different to vitamin K antagonists.

The ROCKET‐AF trial showed that rivaroxaban was at least as effective as warfarin for the prevention of stroke or systemic embolism in a high thromboembolic risk AF population, with a lower risk of fatal and intracranial bleedings.^17^ A post hoc analysis showed that the relative efficacy and safety of rivaroxaban was independent of the presence of HF.^18^

The primary objective of this study was to explore the risk factors associated with HF worsening (measured by hospitalizations and emergency visits because of HF exacerbations) in patients with AF and HF treated with rivaroxaban. In addition, the cut‐off values of quantitative variables associated with HF worsening, the clinical profile of this population and the persistence with rivaroxaban were also analyzed.

## 2 METHODS

This was a multicenter, prospective, observational, cohort study that included adult patients with a diagnosis of nonvalvular AF,^10^ NYHA class I–IV chronic HF (regardless of ejection fraction),^2^ receiving rivaroxaban at least 4 months before being enrolled. By contrast, patients participating in a research program which involved some intervention beyond clinical practice, with significant mitral stenosis or other heart valvular diseases that required specific treatment (prosthesis or valvuloplasty), or with severe cognitive impairment were excluded from the study. The study was approved by the research ethical committee of Parc de Salut Mar. All patients provided written informed consent, before being included.

Patients were consecutively recruited during a routine follow‐up visit between March 2018 and July 2019 in 71 participating centers from Spain (all patients who met the inclusion/exclusion criteria were asked to be included). As the study was based on the routine clinical practice of the management of HF/AF patients, no specific diagnostic or therapeutic action were required for participating. Data about clinical history of the patients were collected from the electronic health records of the patients and in addition the investigator could complement the information by interviewing the patient during the routine visit. Patients were followed‐up during 2 years (baseline, follow‐up visits 1–3, and end of observation), according to clinical practice.

At baseline, biodemographic data, AF data (time since AF diagnosis, type of AF, CHA_2_DS_2_‐VASc score,^19^ HAS‐BLED score^20^), HF data (time since HF diagnosis, NYHA functional class, type of HF,^2^ etiology of HF), vital signs, cardiovascular risk factors, vascular disease, and other comorbidities, as well as concomitant treatments were recorded. In addition, the Barthel Test,^21^ Frail scale,^22^ and Charlson Index^23^ were also calculated.

The information regarding treatment with rivaroxaban along the study, including the previous use of vitamin K antagonists, dosage, medication adherence, and any change during the follow‐up, was also collected. Laboratory parameters (hemoglobin, fasting glucose, glomerular filtration rate, BNP, and NT‐proBNP) were compared at baseline and at study end (24 months).

The variable for the primary objective was the first HF worsening, defined as first HF hospitalization or admission to emergency department due to a HF exacerbation. The factors potentially influencing the primary endpoint were analyzed and included baseline variables regarding demography, health behavior, vital signs, disease history, comorbidities, prior and concomitant treatments, and laboratory parameters. Additionally, the occurrence of death, thromboembolic events, major bleedings,^24^ intracranial bleedings, and fatal hemorrhages were also determined. The event rates during the follow‐up according to the use of rivaroxaban before inclusion (<6 months vs. 6–12 months vs. ≥12 months and <1 year vs. ≥1 year) were calculated.

Three types of analysis population were defined: (1) Safety analysis set: all patients that had received antithrombotic treatment because of AF, with rivaroxaban since at least 4 months before entering the study. The safety analysis set was used for the description of the safety analysis; (2) full analysis set: all patients that had received antithrombotic treatment because of AF, with rivaroxaban since at least 4 months before entering the study and who had satisfied the inclusion/exclusion criteria defined in the study protocol. The full analysis set was used for the main analyses; (3) per protocol set: all patients that had received antithrombotic treatment because of AF, with rivaroxaban since at least 4 months before entering the study, who had satisfied the inclusion/exclusion criteria defined in the study protocol and that had had at least one postbaseline visit, except for premature terminations due to death or adverse events. The per protocol set was used for the baseline description and the analyses of the primary and secondary objectives.

### 2.1 Statistical analysis

For the descriptive analyses, absolute and relative frequency distributions were used for the qualitative variables, and measures of central tendency (mean) and dispersion (standard deviation) for quantitative variables. Categorical variables were compared with the *χ* ^2^ test or the Fisher exact test when appropriate. When two independent means were compared, the *t* student test was used. The evolution (study end‐baseline) of laboratory parameters were compared using the paired sample *t* test.

Kaplan–Meier curves were used to assess the time to the first clinical outcome (HF worsening episode, thromboembolic event, all‐cause death, and major bleeding) according to the previous use of rivaroxaban (<6 months vs. 6–12 months vs. ≥12 months and <1 year vs. ≥1 year) and the Log Rank (Mantel–Cox) was calculated for each comparison to determine the presence of statistical differences.

To explore the risk factors for first HF hospitalization/emergency visit (HF worsening episode), baseline variables, including demography, health behavior, vital signs, disease history, comorbidities, laboratory parameters, prior and concomitant treatments, and previous hospitalizations and admissions to the emergency department, were considered for inclusion in a Cox proportional hazard model. The Cox model was computed by considering only the first event (i.e., hospitalization/emergency visit) after the baseline visit. To assess the cut‐off values of quantitative variables associated with HF worsening, they were transformed into dichotomous variables according to the cut‐offs defined in the literature.^14^, ^15^ Initially, feasibility of the factors was explored using bivariate models. Then, those with a *p* < .15 were included in the multivariate models. Only the significant factors (*p* < .05) were finally considered to build the models. All analyses are performed with SAS® version 9.4 (SAS Institute, Inc.).

## 3 RESULTS

**Figure 1:**
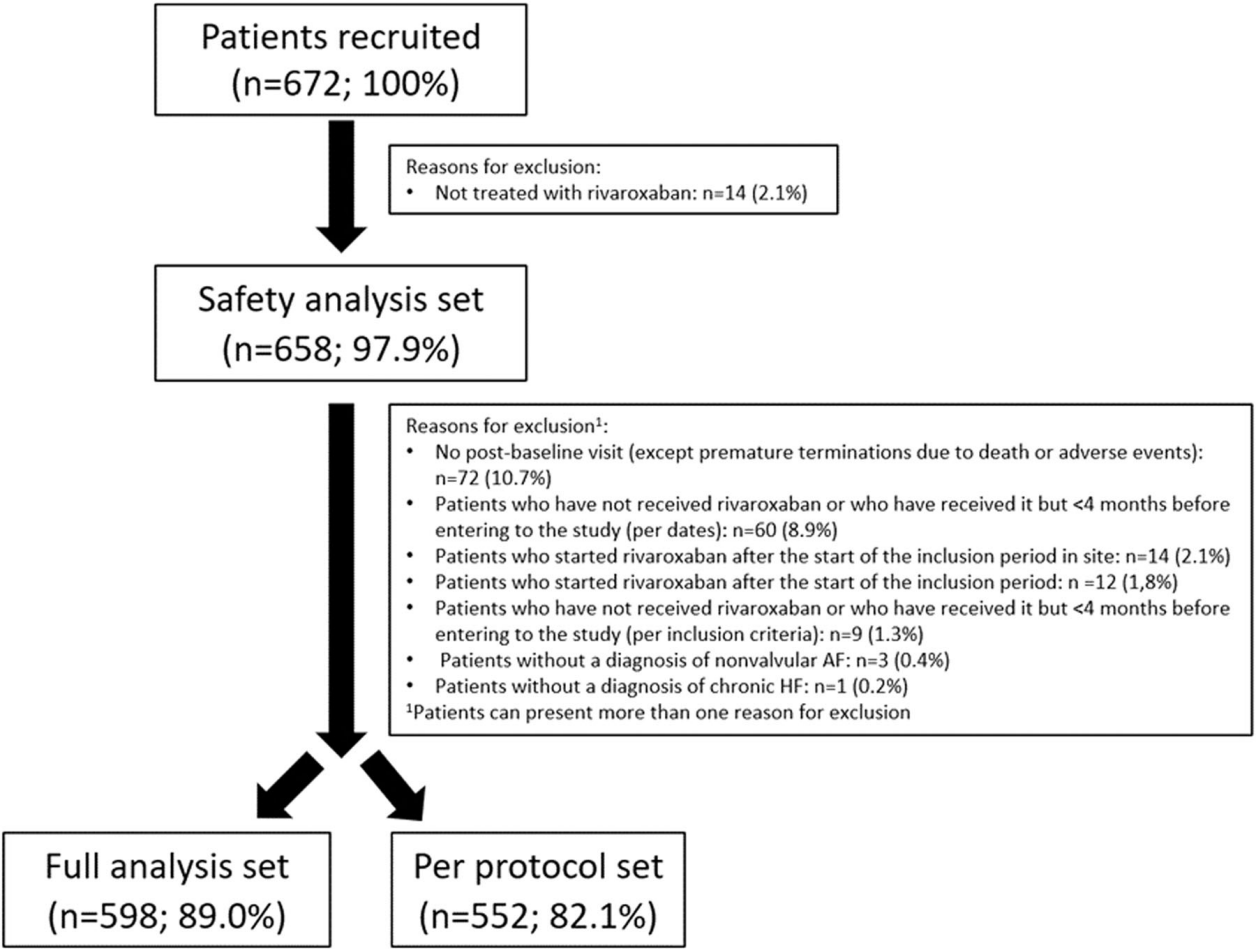
Flow chart of the study.

A total of 672 patients were recruited, of whom 658 (97.9%) patients were included in the safety analysis set, 598 (89.0%) in the full analysis set, and 552 (82.1%) in the per protocol set. Reasons for exclusion are summarized in Figure 1. At baseline, mean age was 73.7 ± 10.9 years, 64.9% were male, and 33.9% were considered as frail. With regard to AF, 53.9% of patients had permanent AF, mean CHA_2_DS_2_‐VASc was 4.1 ± 1.5 and HAS‐BLED 1.6 ± 0.9. With respect to HF, the majority of patients were on NYHA functional class II (58.7%) or III (23.2%), 51.3% had HF with preserved ejection fraction, and the most common etiologies of HF were hypertensive (28.6%), dilated (27.0%), and ischemic (22.8%). Comorbidities were common, 77.5% had arterial hypertension, 39.1% previous coronary artery disease, 37.3% diabetes and 32.4% chronic kidney disease. With regard to HF treatments, 85.5% were taking a renin angiotensin system inhibitor, 79.7% a beta blocker and 51.4% an aldosterone antagonist (Table 1).

**Table 1:** Baseline clinical characteristics of the study population, per protocol set (*n* = 552).

Biodemographic data	
Age, years	73.7 ± 10.9
<65 years, *n* (%)	118 (21.4)
≥65–≤75 years, *n* (%)	163 (29.5)
>75 years, *n* (%)	271 (49.1)
Gender (male), *n* (%)	358 (64.9)
Barthel test	94.6 ± 12.4
Frail scale	1.5 ± 1.3
Frail (score > 0), *n* (%)	113 (33.9)
Charlson Index	2.0 ± 1.1

*AF data*	
Time since AF diagnosis, months	58.8 ± 59.7
Type of AF, *n* (%)	
Paroxysmal	170 (31.1)
Persistent	65 (11.9)
Long‐standing persistent	17 (3.1)
Permanent	295 (53.9)
Missing	5
CHA_2_DS_2_‐VASc score	4.1 ± 1.5
HAS‐BLED score	1.6 ± 0.9

*HF data*	
Time since HF diagnosis, months	47.1 ± 50.2
NYHA functional class, *n* (%)	
Class I	96 (17.4)
Class II	324 (58.7)
Class III	128 (23.2)
Class IV	4 (0.7)
Department where the patient was visited, *n* (%)	
Cardiology	463 (83.9)
Internal medicine	82 (14.9)
Other	7 (1.2)
HF unit	272 (49.3)
HF classification, *n* (%)	
HF with reduced ejection fraction	173 (31.3)
HF with mildly reduced ejection fraction	96 (17.4)
HF with preserved ejection fraction	283 (51.3)
Etiology of HF, *n* (%)	
Hypertensive	158 (28.6)
Dilated	149 (27.0)
Ischemic	126 (22.8)
Valvulopathy	35 (6.3)
Hypertrophic	18 (3.3)
Toxics	10 (1.8)
Restrictive or infiltrative	8 (1.5)
Missing	48 (8.7)

*Physical examination*	
Body mass index (Kg/cm^2^)	28.8 ± 5.3
Heart rate (bpm)	71.6 ± 14.7
Systolic blood pressure (mmHg)	125.2 ± 18.9
Diastolic blood pressure (mmHg)	72.8 ± 11.6

*Cardiovascular risk factors*	
Arterial hypertension, *n* (%)	428 (77.5)
Hyperlipidemia, *n* (%)	302 (54.7)
Diabetes mellitus, *n* (%)	206 (37.3)
Smoking, *n* (%)	
No	492 (89.1)
Current smoker	27 (45.0)
Recent ex‐smoker (<1 year)	14 (23.3)
Former ex‐smoker (>1 year)	19 (31.7)

*Vascular disease*	
Previous coronary artery disease, *n* (%)	152 (39.1)
Previous cerebrovascular disease, *n* (%)	69 (12.5)

Other comorbidities	
Chronic kidney disease, *n* (%)	179 (32.4)
Chronic obstructive pulmonary disease	110 (19.9)
Cancer	59 (10.7)
Nonsevere dementia	20 (3.6)
Liver dysfunction	5 (0.9)

Other treatments	
Compliance with diet recommendations, *n* (%)	474 (85.9)
Compliance with pharmacological treatments for HF, *n* (%)	546 (98.9)
Diuretics, *n* (%)	499 (90.6)
RAAS inhibitors, *n* (%)	471 (85.5)
Angiotensin converting enzyme inhibitors, *n* (%)	202 (36.7)
Angiotensin II receptor blockers, *n* (%)	131 (23.8)
Sacubitril/valsartan, *n* (%)	138 (25.0)
Beta blockers, *n* (%)	439 (79.7)
Mineralocorticoid receptor antagonists, *n* (%)	283 (51.4)
Digoxin, *n* (%)	127 (23.0)
Ivabradine, *n* (%)	17 (3.1)
Antiarrhythmics, *n* (%)	108 (19.6)
Amiodarone	81 (14.7)
Flecainide	18 (3.3)
Dronedarone	3 (0.5)
Propafenone	3 (0.5)
Sotalol	3 (0.5)
Antiplatelets, *n* (%)	71 (12.9)
Implantable cardioverter‐defibrillator, *n* (%)	77 (19.8)
Pacemaker, *n* (%)	54 (13.9)
Resynchronization, *n* (%)	40 (10.3)
Previous ablation, *n* (%)	36 (9.2)

Abbreviations: AF, atrial fibrillation; CrCl, creatinine clearance; HF, heart failure; INR, international normalized ratio; NYHA, New York Heart Association; RAAS, renin angiotensin system inhibitors; VKA, vitamin K antagonists.

Mean time from start of treatment to study entering was 25.5 ± 18.8 months (<6 months: 12.6%, 6–12 months: 21.1%, ≥12 months: 66.3%; <1 year: 33.7%, ≥1 year: 66.3% of the study population). 69.0% of patients were taking rivaroxaban 20 mg and the remaining 31.0% rivaroxaban 15 mg. After 2 years of follow‐up, only 6.9% permanently discontinued rivaroxaban treatment (excluding patients who died), mainly because of bleeding or severe worsening of renal function, 8.5% temporarily interrupted the treatment and in 13.0% of patients, the dose of rivaroxaban was modified. In the safety population (*n* = 658), only 3.0% of patients presented a serious adverse event related to rivaroxaban (Table 2).

**Table 2:** Treatment with rivaroxaban during the study, per protocol set (*n* = 552).

Time from start of treatment to study entering, months	25.5 ± 18.8
Previous VKA, *n* (%)	248 (44.9)
Labile INR, *n* (%)	183 (73.5)
Dose, *n* (%)	
15 mg	171 (31.0%)
20 mg	381 (69.0%)
Permanent discontinuation, *n* (%)	38 (6.9)
Reasons for rivaroxaban withdrawal, *n* (%)	
Hemorrhagic events	13 (34.2)
Advanced kidney disease	11 (28.9)
Others	14 (36.8)
Any change with rivaroxaban since the beginning of the study,^a^ *n* (%)	109 (19.8)
Dose adjustment, *n* (%)	72 (13.0)
Temporary interruption, *n* (%)	47 (8.5)

^a^ Patient may present more than one option.

After 24 months of follow‐up, whereas hemoglobin and NT‐pro‐BNP values remained stable, there was a significant decrease of glomerular filtration rate and BNP levels (Table 3). With regard to outcomes, 11.6% of patients died during the follow‐up, 2.9% had a thromboembolic event, 3.1% a major bleeding, 0.5% an intracranial bleeding and no patient had a fatal hemorrhage. In addition, 24.9% of patients developed HF worsening (hospitalization of visit to the emergency department). No significant differences were observed in the event rates according to the use of rivaroxaban before inclusion: <6 months versus 6–12 months versus ≥12 months or <1 year versus ≥1 year (Supporting Information S1: Tables 1 and 2). Additionally, the Kaplan–Meier curves confirmed these results (Supporting Information S1: Figures 1–4).

**Table 3:** Evolution of laboratory parameters, per protocol set (*n* = 552).

	Baseline	Study end (24 months)	*p* Value
Hemoglobin (g/dL)	13.4 ± 1.9	13.5 ± 2.0	.39
Fasting glucose (mg/dL)	115.2 ± 38.6	115.3 ± 36.2	.96
Glomerular filtration rate (mL/min per 1.73 m^2^)	62.0 ± 20.2	58.5 ± 19.8	.004
BNP (pg/L)	708.7 ± 1329.9	312.5 ± 288.7	<.01
NT‐proBNP (pg/L)	2771.2 ± 3458.8	2527.1 ± 4800.6	.33

Cut‐off values of quantitative variables associated with HF worsening were analyzed, and these included low diastolic blood pressure, renal dysfunction, and anemia (Table 4). In addition, the multivariate analysis showed that increasing age, the history of chronic obstructive pulmonary disease, the previous use of vitamin K antagonists, and restrictive or infiltrative cardiomyopathies were independently associated with HF worsening (Table 4). Low diastolic blood pressure, defined as <75 mmHg was reported in 54.3% of patients. Compared to patients with diastolic blood pressure ≥75 mmHg, those with <75 mmHg were older, more fragile, and had more prior coronary artery disease and chronic kidney disease, with a trend towards more HF with reduced ejection fraction (Supporting Information S1: Table 3).

**Table 4:** Cutoff values of quantitative variables associated with HF worsening and independent factors associated with HF worsening.

Variable	HR (95% CI)	*p* Value
**Cutoff values of quantitative variables associated with HF worsening**		
DBP < 75 vs. ≥75 mmHg	1.67 (1.18.2.38)	.004
eGFR <60 vs ≥60 mL/min/1.73 m^2^	1.67 (1.17.2.39)	.005
Hemoglobin <13 vs. ≥13 g/dL	1.61 (1.15.2.27)	.006
Age <80 vs. ≥80 years	0.52 (0.37.0.73)	.0001
Age <75 vs. ≥75 years	0.49 (0.35.0.70)	<.0001
**Independent factors associated with HF worsening**		
Age (years), per each unit of the variable	1.03 (1.01–1.05)	.004
COPD	2.08 (1.36–3.17)	.001
Previous use of VKA	1.78 (1.21–2.63)	.004
Etiology of HF diagnosis		
Restrictive cardiomyopathy vs. others	9.24 (2.21–38.62)	.002
Infiltrative cardiomyopathy vs. others	6.03 (2.17–16.76)	.001

Abbreviations: CI, confidence interval; COPD, chronic obstructive pulmonary disease; DBP, diastolic blood pressure; eGFR, estimated glomerular filtration rate; HR, hazard ratio; VKA, vitamin K antagonists.

## 4 DISCUSSION

This study identified in a wide sample of patients with AF and HF treated with rivaroxaban, independent predictors of worsening HF episodes. Increasing age, previous chronic obstructive pulmonary disease, previous treatment with vitamin K antagonists and HF etiology. After 2 years of follow‐up, nearly 25% of patients developed a HF worsening episode, 12% of patients died, and thromboembolic and bleeding events were low.

In our study, patients were old, had many comorbidities, a high thromboembolic risk and approximately one‐third were considered as frail. In the subgroup of patients with HF included in the ROCKET‐AF trial, median age was 72 years, CHA_2_DS_2_‐VASc 5.1 and one‐third presented HF with reduced ejection fraction.^17^ The GLORIA‐AF registry included newly diagnosed patients with AF and CHA_2_DS_2_‐VASc score ≥1. In this study, 24% of patients had HF, of whom 41% were ≥75‐year‐old, CHA_2_DS_2_‐VASc score was 3.9% and 38% had HF with reduced ejection fraction.^25^ In summary, patients with HF and AF are usually elderly, with a significant burden of comorbidities, and share etiopathogenic and risk factors.^26^ Therefore, our sample was representative of real‐life patients with AF and HF. On the other hand, our study provided relevant information of the whole spectrum of HF, regardless of ejection fraction or the etiology of HF, as this study lacked strict selection criteria and may capture the effects of rivaroxaban in the real‐world Spanish setting, supporting the generalizability of the results. This is important, as information regarding some phenotypes of HF, such as those with mildly reduced ejection fraction is very scarce.^27^

Although other registries have analyzed the factors associated with the severity of HF and AF, patients were mainly treated with vitamin K antagonist, and these factors could change when other anticoagulants are taken.^28^, ^29^ In this study all patients were taking rivaroxaban to avoid possible bias when using different anticoagulants, facilitating the focus on the primary endpoint of the study, as this was a homogeneous population. One out of four patients developed a HF worsening episode, defined as first HF hospitalization or visit to the emergency department due to HF decompensation. Comorbidities such as chronic obstructive pulmonary disease were identified as risk factors for HF progression. In this context, it is necessary a comprehensive management of patients with AF and HF, focusing not only on the reduction of thromboembolic events with appropriate anticoagulation, but also on HF complications and comorbidities.^2^, ^10^ The previous use of vitamin K antagonists were also associated with worsening HF. One of the main limitations of these drugs is their great response variability, leading to frequent monitoring of anticoagulant activity and dose adjustments. This is even more marked in HF patients.^30^, ^31^ By contrast, rivaroxaban provides a consistent and stable anticoagulant effect, even in these patients, leading to a good safety profile and a protective effect.^18^ Of note, event rates did nor differ according to the time of use of rivaroxaban before inclusion, emphasizing its safety in clinical practice.

Previous studies performed in HF population have also shown that chronic kidney disease is associated with HF hospitalization.^14^, ^15^ Although renal function decline is common among patients with AF, different studies have shown that compared with warfarin, rivaroxaban has a lower risk of renal function impairment.^32^, ^33^, ^34^ In our study there was a modest reduction of renal function that could be explained by the high risk clinical profile (i.e., elderly patients with HF, AF, and many comorbidities). Although low diastolic blood pressure was associated with HF worsening, the worse clinical profile of these patients could explain at least in part this point.

Approximately 70% of patients were taking rivaroxaban 20 mg and 30% rivaroxaban 15 mg. Although it was not specifically analyzed, considering that around one‐third of patients had chronic kidney disease, these results suggest that in the majority of patients rivaroxaban was properly prescribed. A recent study has shown that in real‐life patients with AF, in less than 10% of patients rivaroxaban is underdosed.^35^ The simplicity of rivaroxaban dosage may have contributed, as it only depends on renal function.^36^ On the other hand, previous studies have also shown the high adherence and persistence with rivaroxaban, mainly related to the low risk of adverse events and its simplicity of use.^37^, ^38^ In our study, only 7% of patients permanently discontinued rivaroxaban.

In our study, after 2 years of follow‐up, nearly 12% of patients died and 3% had a thromboembolic event. Although in nonanticoagulated patients with AF the most important complication is stroke, in anticoagulated patients, mortality is mainly related to other causes different to cerebrovascular disease,^25^, ^39^ indicating the need for a comprehensive approach in the management of patients with HF and AF. Despite the risk of hemorrhage is increased in HF patients,^40^ our study showed that rivaroxaban had a good safety profile in this population, with a low risk of major and intracranial hemorrhages in clinical practice.

This study has some limitations. First, a limitation of the study design might be the delay window between treatments start and study inclusion, at least 4 months after rivaroxaban treatment initiation. This constraint was introduced to prevent any interference on prescription behavior. Therefore, all events occurring during this period accounted for retrospective data, but not for the primary objective. Although this design may challenge the interpretation of the results and limit their scope, it is noteworthy that the purpose of our study was to assess the risk factors encountered in a follow‐up visit during rivaroxaban treatment, rather than those at the moment of treatment start. Second, spectrums of disease severity existed for comorbidities; however, for the purposes of modeling, these diseases were treated as binary events. Third, as patients included in this study were representative of the Spanish population with HF and AF taking rivaroxaban, the results can only be extended to patients with a similar clinical profile.

## 5 CONCLUSION

Approximately one out of four anticoagulated patients with HF and AF developed a HF worsening episode after 2 year of follow‐up. Increasing age, the history of chronic obstructive pulmonary disease, the previous use of vitamin K antagonists, and restrictive or infiltrative cardiomyopathies, were independently associated with HF worsening, suggesting that a comprehensive approach is required to reduce HF burden in this population.

## AUTHOR CONTRIBUTIONS

All authors have contributed significantly to the work presented in this article, contributing to the conception, design, or acquisition of information, or to the analysis and interpretation of data. All the authors have participated in the drafting and/or revision of the manuscript and accept its publication.

## CONFLICT OF INTEREST STATEMENT

The authors received honoraria from Bayer Hispania S.L. for their participation as researchers in the FARAONIC study sponsored by Bayer. Carles Rafols is an employee of Bayer Hispania S.L.

## Supporting information

Supporting information.

## Data Availability

The data that support the findings of this study are available from the corresponding author upon reasonable request.
