# Supplementary material for: Factors associated with disease progression in patients with atrial fibrillation and heart failure anticoagulated with rivaroxaban
Source: Clin Cardiol. 2023 Nov 29;47(2):e24189. doi: 10.1002/clc.24189 (PMC10823448; doi:10.1002/clc.24189)

**Supplementary table 1. Event rates after 24 months of follow-up according to the use of rivaroxaban before inclusion (<6 months, 6-12 months and ≥12 months).**

|  | **Overall** | **<6 months (12.6%)** | **6-12 months (21.1%)** | **≥12 months (66.3%)** | **P** |
| --- | --- | --- | --- | --- | --- |
| **HF worsening, %** | 24.9 | 30.8 | 25.2 | 23.7 | 0.58 |
| **TE events, %** | 2.9 | 2.3 | 3.4 | 3.1 | 0.89 |
| **Death, %** | 11.6 | 11.2 | 10.1 | 12.3 | 0.90 |
| **Major bleeding, %** | 3.1 | 4.6 | 3.7 | 2.9 | 0.56 |

HF: heart failure; TE: thromboembolic.

**Supplementary table 2. Event rates after 24 months of follow-up according to the use of rivaroxaban before inclusion (<1 year and ≥1 year).**

|  | **Overall** | **<1 year (33.7%)** | **≥1 year (66.3%)** | **P** |
| --- | --- | --- | --- | --- |
| **HF worsening, %** | 24.9 | 27.3 | 23.7 | 0.49 |
| **TE events, %** | 2.9 | 2.8 | 3.1 | 0.99 |
| **Death, %** | 11.6 | 10.6 | 12.3 | 0.85 |
| **Major bleeding, %** | 3.1 | 4.0 | 2.9 | 0.35 |

HF: heart failure; TE: thromboembolic.

**Supplementary table 3. Baseline clinical characteristics of the study population according to diastolic blood pressure (<75 mmHg vs ≥75 mmHg).**

|  | **Overall** | **DBP <75 mmHg (54.3%)** | **DBP ≥75 mmHg (45.7%)** | **P** |
| --- | --- | --- | --- | --- |
| **Biodemographic data** | | | | |
| Age, years | 73.7±10.9 | 74.7±11.0 | 72.5±10.6 | 0.009 |
| Gender (male), % | 64.9 | 65.7 | 63.9 | 0.63 |
| Barthel Test | 94.6±12.4 | 93.3±13.7 | 95.3±12.4 | 0.06 |
| Frail scale | 1.5±1.3 | 1.7±1.4 | 1.3±1.2 | <0.01 |
| Charlson Index | 2.0±1.1 | 2.1±1.1 | 2.0±1.0 | 0.37 |
| **AF data** | | | | |
| Type of AF, %  Paroxysmal  Persistent  Long-standing persistent  Permanent | 31.1  11.9  3.1  53.9 | 32.2  11.1  2.2  54.5 | 30.8  13.2  3.6  52.4 | 0.45 |
| CHA_2_DS_2_-VASc score | 4.1±1.5 | 4.1±1.6 | 4.1±1.5 | 0.70 |
| HAS-BLED score | 1.6±0.9 | 1.7±1.0 | 1.5±0.9 | 0.14 |
| Rivaroxaban 20 mg, % | 69.0 | 61.4 | 76.1 | <0.01 |
| **HF data** | |  |  |  |
| NYHA functional class, %  Class I  Class II  Class III  Class IV | 17.4  58.7  23.2  0.7 | 18.7  55.4  25.1  0.8 | 16.2  61.2  21.9  0.7 | 0.44 |
| HF classification, %  HFrEF  HFmrEF  HFpEF | 31.3  17.4  51.3 | 34.0  15.9  50.1 | 27.2  20.5  52.3 | 0.06 |
| **Cardiovascular risk factors/Vascular disease** | | | | |
| Arterial hypertension, % | 77.5 | 72.4 | 83.1 | 0.004 |
| Diabetes mellitus, % | 37.3 | 37.0 | 37.5 | 0.51 |
| Previous CAD, % | 39.1 | 42.3 | 34.8 | <0.01 |
| Previous stroke, % | 12.5 | 11.4 | 14.6 | 0.26 |
| Chronic kidney disease, % | 32.4 | 38.7 | 26.5 | 0.002 |

AF: atrial fibrillation; CAD: coronary artery disease; DBP: diastolic blood pressure; HF: heart failure; HFmrEF: heart failure with mildly reduced ejection fraction; HFpEF: heart failure with preserved ejection fraction; HFpEF: heart failure with preserved ejection fraction; NYHA: New York Heart Association.

**Supplementary figure 1. Kaplan-Meier survival curves of heart failure worsening according to the use of rivaroxaban before inclusion**

**A. Use of rivaroxaban <1 year and ≥1 year before inclusion**


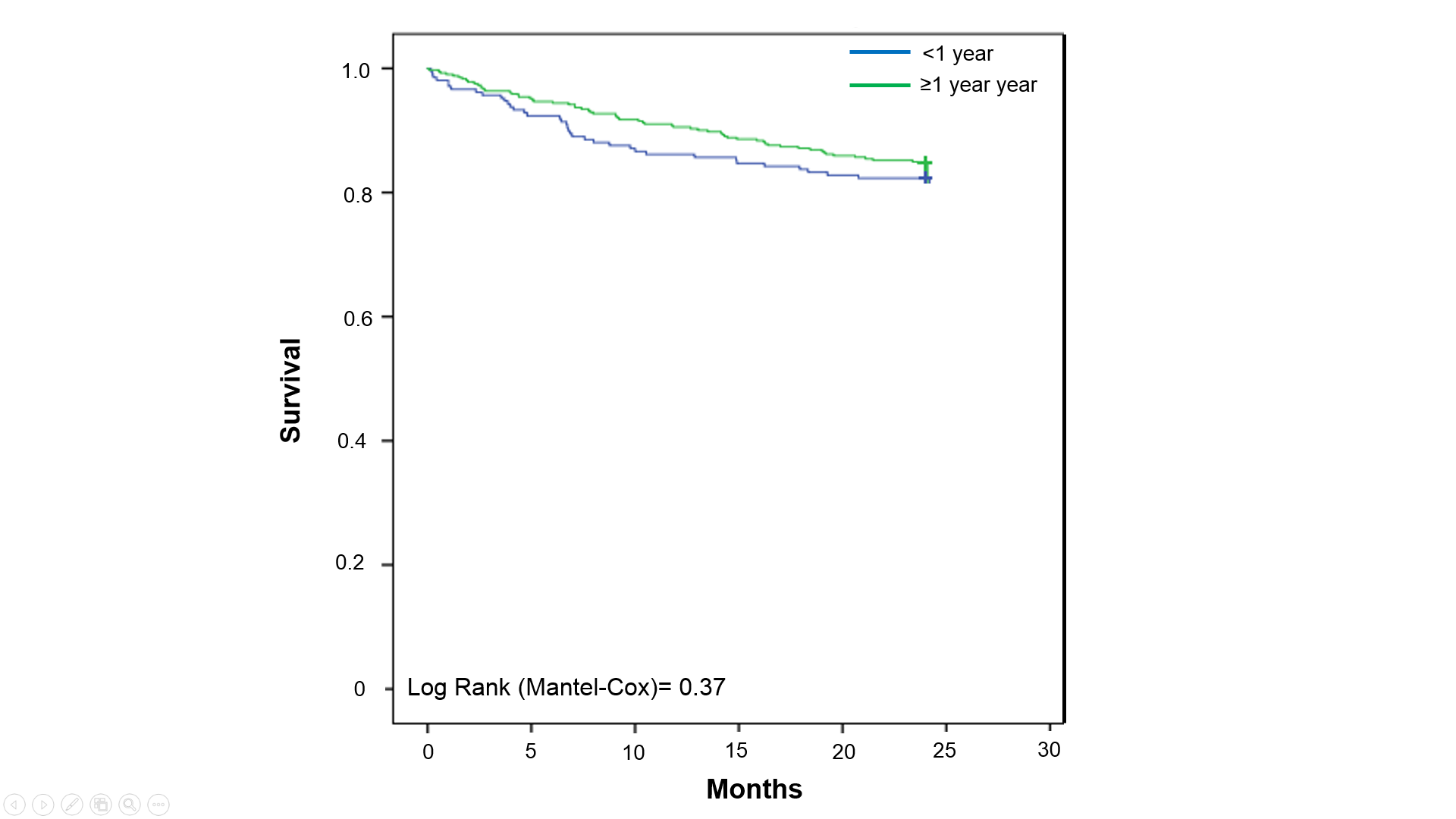


**B. Use of rivaroxaban <6 months, 6-12 months and ≥12 months before inclusion.**


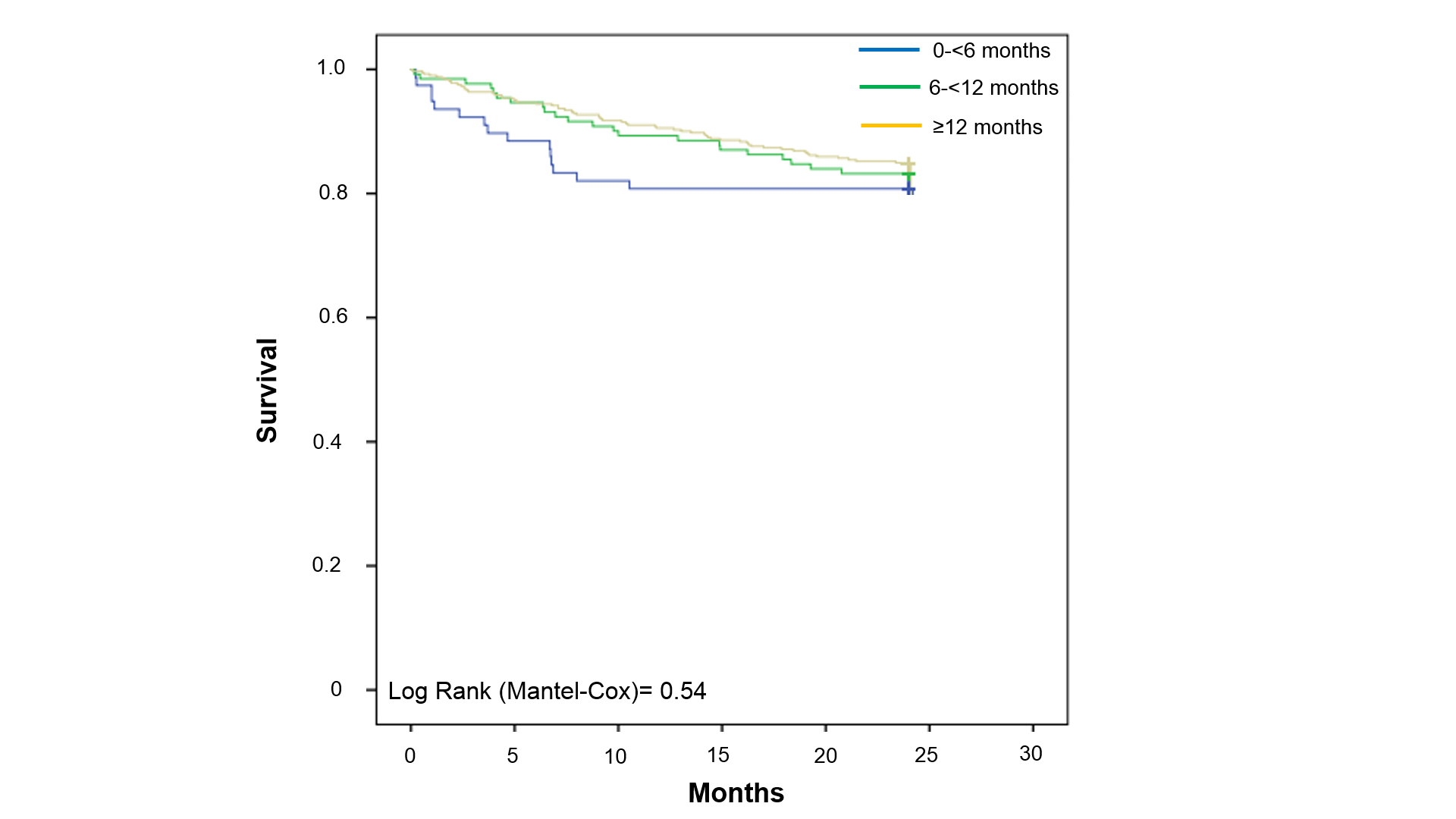


**Supplementary figure 2. Kaplan-Meier survival curves of thromboembolic event according to the use of rivaroxaban before inclusion**

**A. Use of rivaroxaban <1 year and ≥1 year before inclusion**


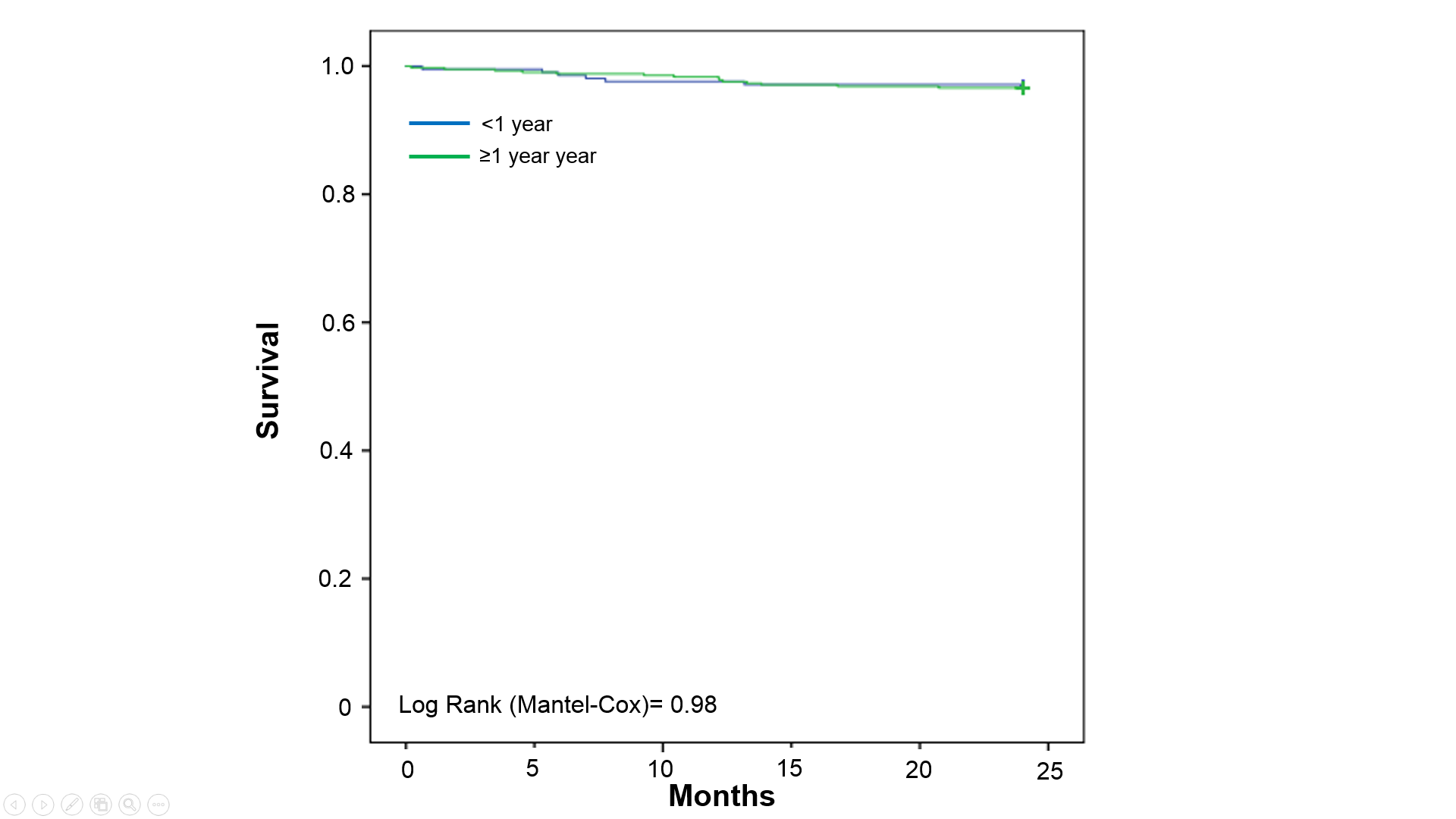


**B. Use of rivaroxaban <6 months, 6-12 months and ≥12 months before inclusion.**


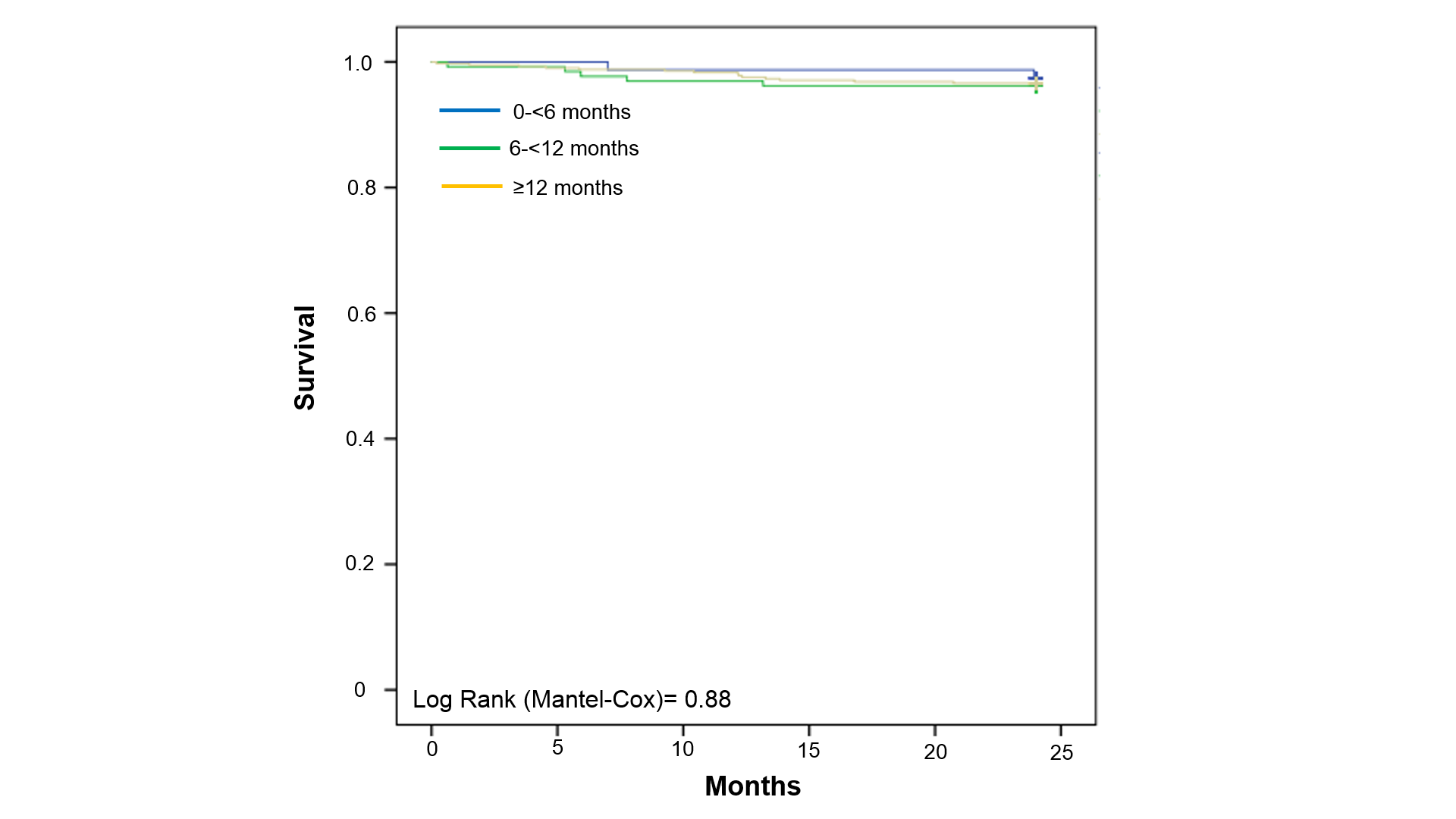


**Supplementary figure 3. Kaplan-Meier survival curves of all-cause death according to the use of rivaroxaban before inclusion**

**A. Use of rivaroxaban <1 year and ≥1 year before inclusion**


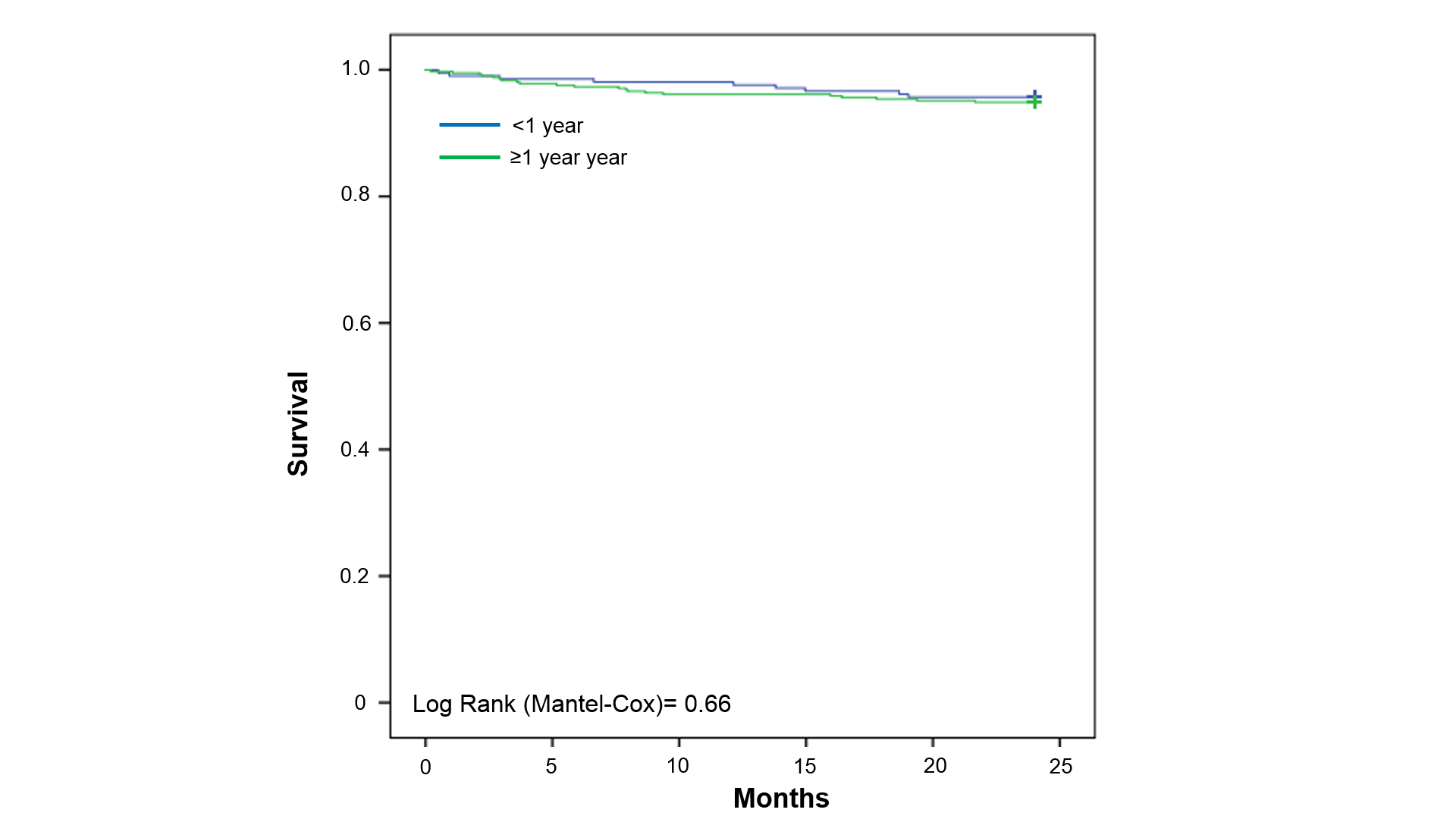


**B. Use of rivaroxaban <6 months, 6-12 months and ≥12 months before inclusion.**


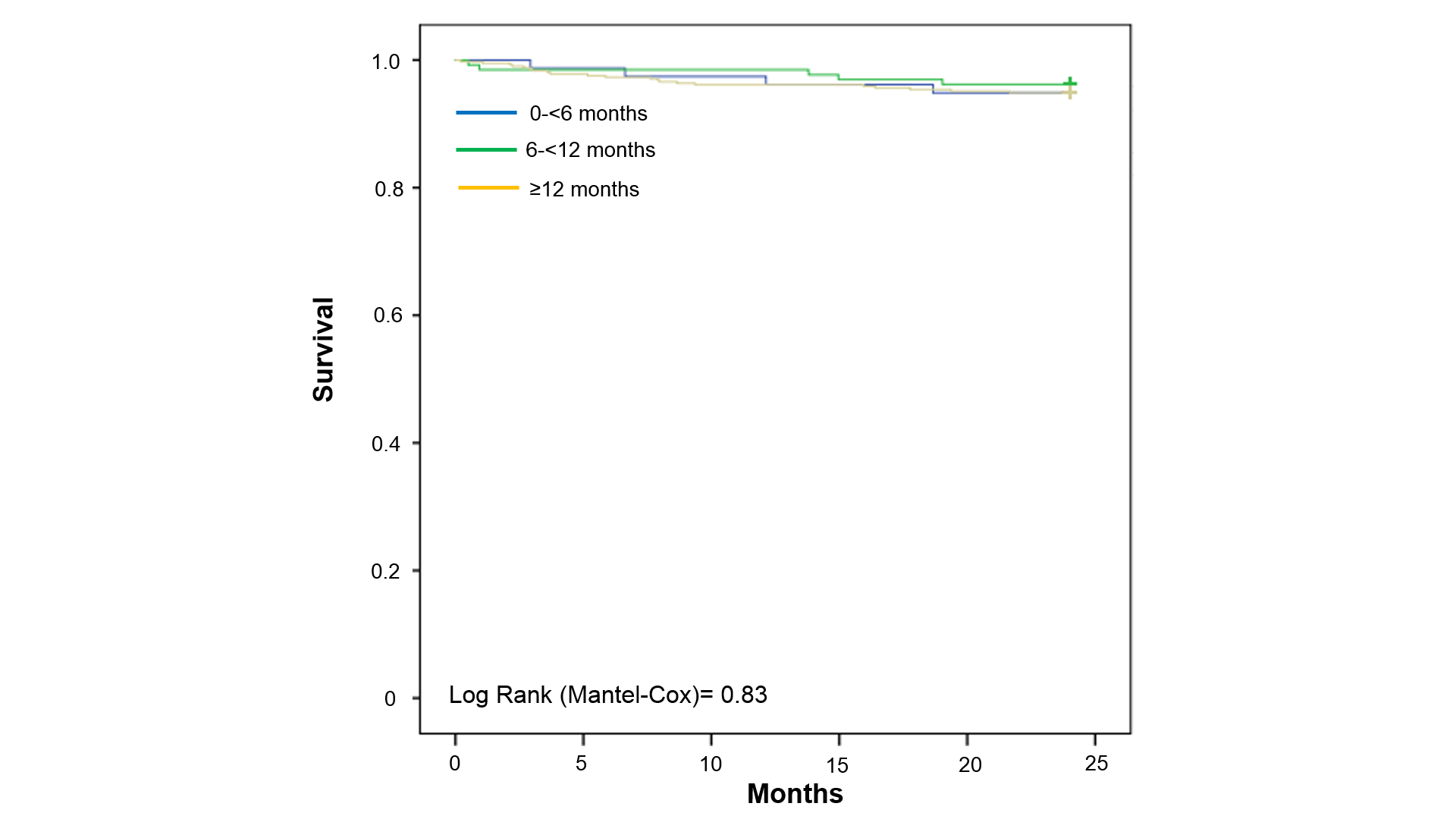


**Supplementary figure 4. Kaplan-Meier survival curves of major bleeding according to the use of rivaroxaban before inclusion**

**A. Use of rivaroxaban <1 year and ≥1 year before inclusion**


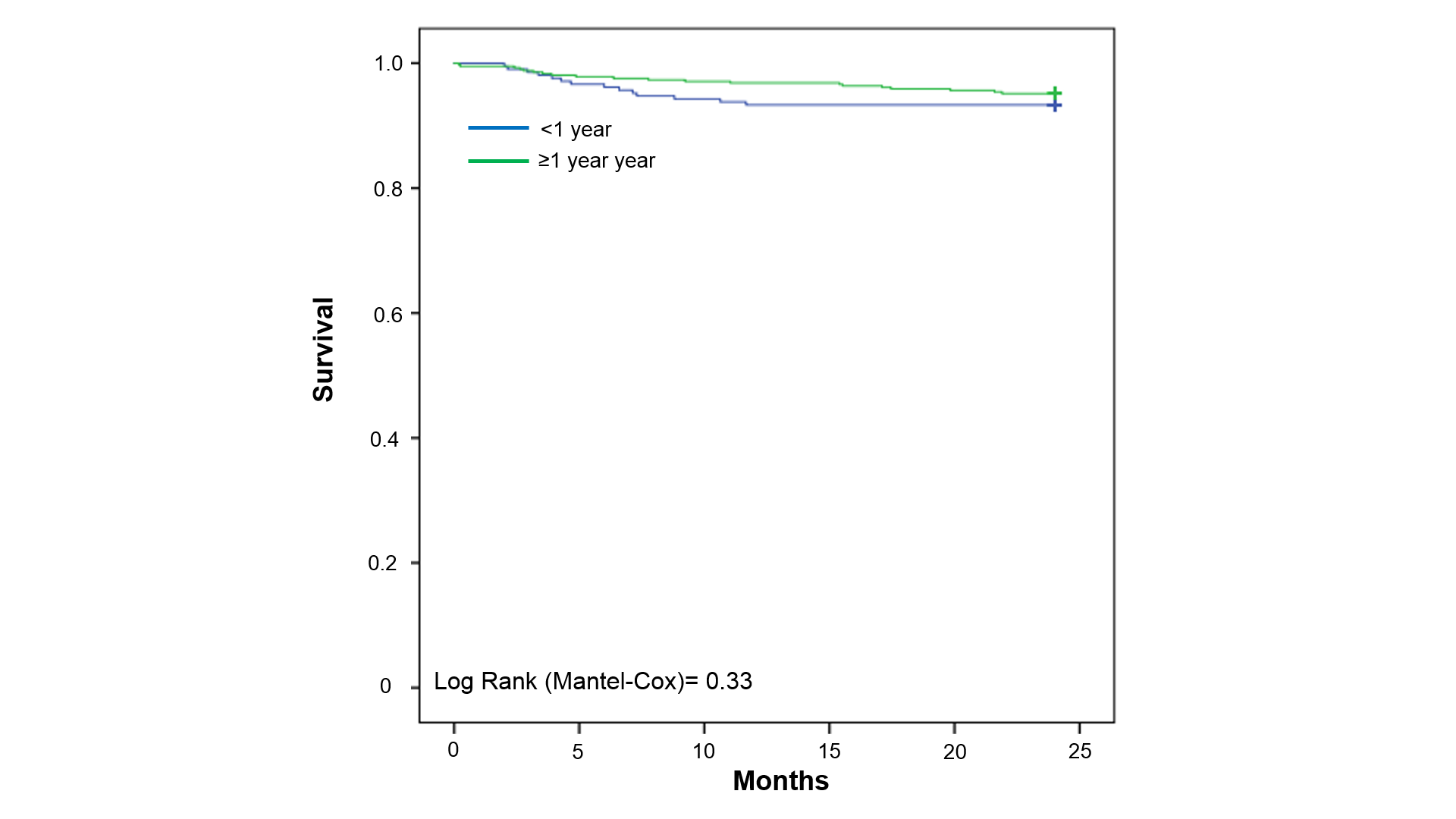


**B. Use of rivaroxaban <6 months, 6-12 months and ≥12 months before inclusion.**


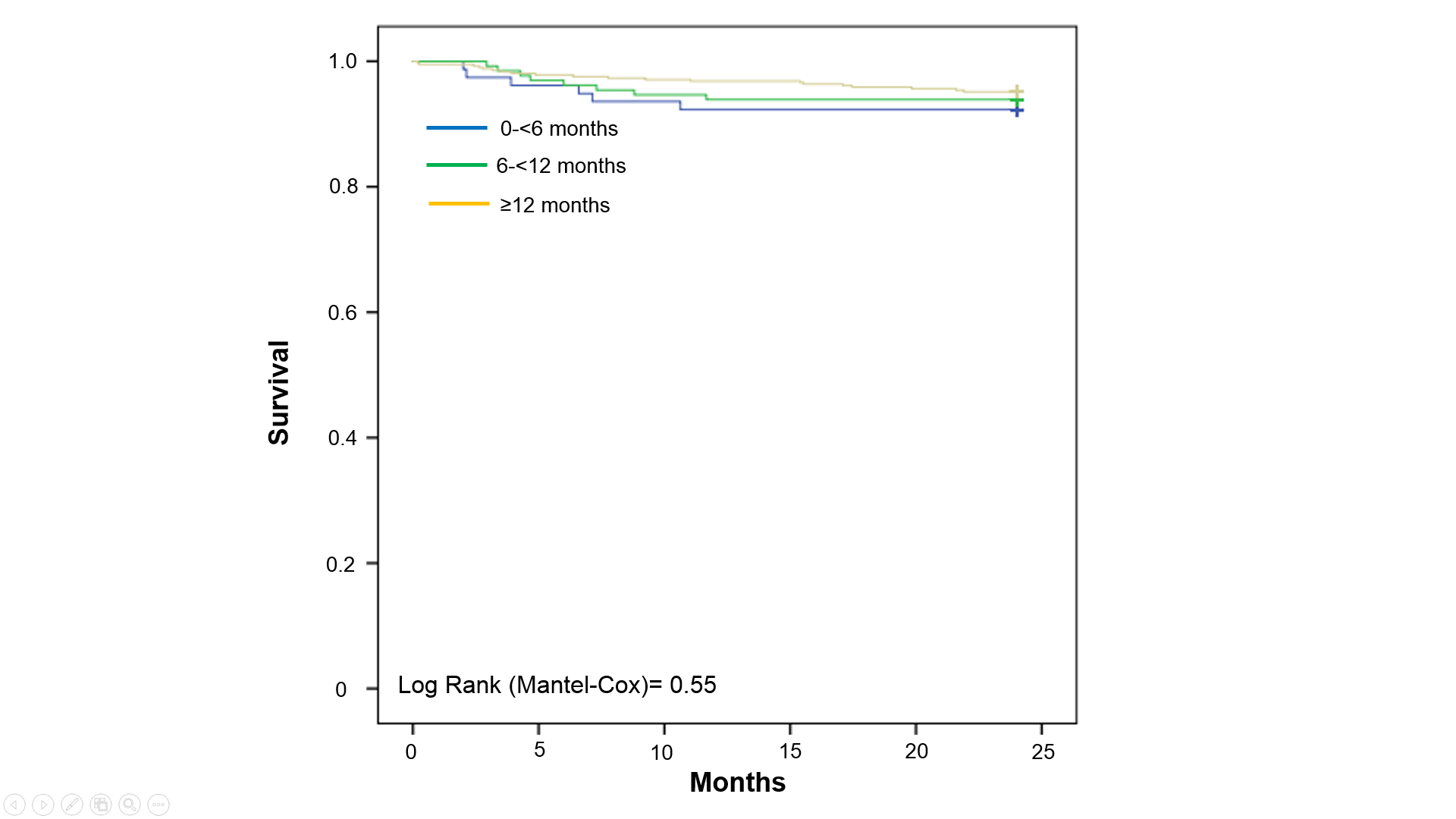

Supplement: Supplementary file 1 — Supporting information. [file CLC-47-e24189-s001.docx]
